# Exercise-Induced Extracellular Vesicle-Associated miRNAs in Cognitive Aging of Older Adults: A Narrative Review

**DOI:** 10.3390/ijms27198677

**Published:** 2026-09-28

**Authors:** Paulina Małkowska, Magdalena Sochacka, Patrizia Proia, Marek Sawczuk

**Affiliations:** 1 Department of Physiology in Health Sciences, Faculty of Health Sciences, Pomeranian Medical University, 71-210 Szczecin, Poland; paulina.malkowska@pum.edu.pl (P.M.); magdalena.sochacka@usz.edu.pl (M.S.); 2 Institute of Physical Culture Sciences, University of Szczecin, 70-240 Szczecin, Poland; 3 Department of Psychology, Educational Science and Human Movement, University of Palermo, 90133 Palermo, Italy; patrizia.proia@unipa.it; 4 Department of Molecular Biology, Faculty of Physical Culture, Gdansk University of Physical Education and Sport, 80-336 Gdansk, Poland

**Keywords:** extracellular vesicles, microRNAs, cognitive aging, neuroinflammation, microglia, inflammaging, brain health, biomarkers, physical activity

## Abstract

Cognitive aging involves chronic neuroinflammation, microglial dysregulation, vascular dysfunction, oxidative stress, and impaired neuroplasticity, and physical activity is one of the few modifiable interventions known to influence all of these processes simultaneously. The molecular mechanisms linking peripheral exercise adaptations to the aging brain, however, remain incompletely defined. This review examines exercise-responsive, EV-associated miRNAs as candidate mediators of this communication across four mechanistic domains: inflammatory signaling (miR-146a, miR-155, miR-223), microglial regulation (miR-124), neurotrophic and synaptic signaling (miR-132, miR-206), and vascular/blood–brain barrier function (miR-126). For each candidate, we distinguish evidence obtained by direct demonstration of an exercise-responsive, EV-associated change from evidence obtained by triangulating separate, non-vesicular, or non-exercise studies—a distinction largely absent from the existing literature. We also evaluate EV-miRNAs as candidate biomarkers of individual exercise responsiveness, separating analytical validity and biological association, for which evidence exists, from predictive validity and clinical utility, for which it does not. Standardized EV characterization, longitudinal exercise interventions, and integration with neuroinflammatory, neurovascular, and cognitive outcomes are needed before these candidates can be considered validated biomarkers of brain health in aging.

## 1. Introduction: Cognitive Aging and the Need for Molecular Prevention Strategies

Population aging is associated with a gradual increase in the prevalence of age-related cognitive decline, mild cognitive impairment, and dementia, which together represent major clinical, social, and public health challenges. Cognitive aging can be defined as the multidimensional pattern of age-related changes in cognitive abilities and the neural system that supports them across adulthood. It includes not only decline, but also relative stability and, in selected domains, preserved performance, and should therefore be distinguished from pathological cognitive impairment and dementia. It is a highly heterogeneous process: whereas some older adults maintain relatively stable cognitive function, others show progressive reductions in episodic memory, executive functions, processing speed, attentional control, and cognitive flexibility. This heterogeneity reflects the interaction of multiple determinants acting across the life course, including genetic and developmental factors, age-related changes in brain structure and connectivity, vascular and metabolic health, neuroinflammation, oxidative stress, sensory function, sleep, mood, educational attainment, cognitively stimulating activity, social engagement, diet, and physical activity [1,2]. Several complementary models have been proposed to explain how these determinants translate into cognitive outcomes in later life. The maintenance–reserve–compensation framework distinguishes brain maintenance, referring to relative preservation of neural resources; cognitive reserve, referring to the flexible and efficient use of available resources; and compensation, referring to the recruitment of additional or alternative neural processes when age-related changes threaten performance [3]. Neuroimaging-based models describe characteristic patterns of such adaptation. The HAROLD model (Hemispheric Asymmetry Reduction in Older Adults) proposes that prefrontal activation becomes less lateralized with age, a pattern that may in some circumstances reflect compensatory bilateral recruitment [4]. The PASA model (Posterior–Anterior Shift in Aging) describes reduced posterior activity accompanied by greater frontal engagement during cognitive tasks [5]. The CRUNCH model (Compensation-Related Utilization of Neural Circuits Hypothesis) further proposes that older adults recruit additional neural resources at lower levels of task demand, but may reach their neural resource ceiling earlier as task complexity increases [6]. Finally, the revised Scaffolding Theory of Aging and Cognition (STAC-r) integrates structural and functional brain changes with compensatory scaffolding and life-course influences, emphasizing that enrichment factors can support, whereas adverse exposures can deplete, neural resources over time [7]. These models are not mutually exclusive; together they emphasize that cognitive aging reflects a dynamic balance between neural deterioration, preservation, reserve, and adaptive reorganization. This conceptual perspective also helps explain why chronological age alone is insufficient to predict cognitive outcome. Biological aging, multimorbidity, cardiometabolic and cerebrovascular burden, inflammatory status, and accumulated lifestyle exposures can modify the relationship between age-related brain changes and observable cognitive performance [3]. From a preventive perspective, cognitive aging should not, therefore, be regarded as an entirely inevitable or exclusively biologically determined process. Although chronological age remains one of the strongest risk factors for cognitive impairment and dementia, a substantial proportion of dementia risk is associated with modifiable factors acting across the life course. The 2024 Lancet Commission report on dementia prevention, intervention, and care emphasizes the importance of multidimensional preventive strategies and identifies lifestyle-related factors, including physical activity, as important targets for reducing dementia risk at the population level [8]. Similarly, the World Health Organization guidelines on risk reduction in cognitive decline and dementia indicate physical activity as one of the key non-pharmacological strategies for adults with normal cognitive functioning and for individuals at increased risk of cognitive deterioration [9]. Physical activity is particularly relevant in the context of brain aging because it exerts multidirectional effects on biological systems involved in cognitive decline. Regular exercise improves cardiovascular and metabolic function, supports cerebrovascular health, modulates chronic low-grade inflammation, enhances neurotrophic signaling, and contributes to the maintenance of muscle function and functional independence in older adults [10,11,12]. These adaptations are especially important for the aging brain, as vascular dysfunction, insulin resistance, chronic inflammation, oxidative stress, and reduced neuroplasticity are among the mechanisms associated with cognitive decline and neurodegenerative processes.

Evidence from systematic reviews and meta-analyses indicates that physical activity-based interventions may improve cognitive function in adults over 50 years of age, although the magnitude of the effect depends on exercise modality, intensity, intervention duration, baseline cognitive status, and the health characteristics of the study population. Aerobic training, resistance training, multicomponent exercise, and Tai Chi were associated with improvements in cognitive function in older adults, supporting the concept of structured physical activity as an accessible and scalable intervention for maintaining cognitive health [13]. More recent evidence suggests that the association between physical activity and the rate of cognitive decline appears to be moderate at the individual level but relevant from a population health perspective, particularly when physical activity is maintained over the long term and incorporated into broader preventive strategies [14]. However, translating this population-level lifestyle guidance into individualized prevention requires identifying the specific molecular pathways through which physical activity acts on the brain, so that intervention intensity, modality, and timing can eventually be tailored to a person’s biological rather than just their chronological profile. This molecular perspective is the focus of the present review.

Despite the growing body of evidence indicating a beneficial association between physical activity and cognitive and brain health, the molecular mechanisms mediating these effects remain incompletely understood. Physical exercise induces a coordinated systemic response involving skeletal muscle, adipose tissue, the cardiovascular system, the immune system, and the central nervous system. This response includes the release of myokines, metabolites, hormones, inflammatory mediators, and extracellular vesicles, which may participate in exercise-induced inter-organ communication [12]. In this context, extracellular vesicle-associated microRNAs represent a promising group of molecular mediators, as they regulate gene expression in target cells and influence inflammatory pathways, synaptic plasticity, oxidative stress responses, and neuronal homeostasis. A better understanding of these relationships may provide new insight into the mechanisms through which physical activity supports cognitive resilience, while also enabling the identification of biomarkers of training responsiveness and potential therapeutic targets in older adults.

This narrative review is based on a non-systematic literature search performed in PubMed, Scopus, and Web of Science, using combinations of the terms “extracellular vesicles”, “exosomes”, “microRNA”, “exercise”, “physical activity”, “cognitive aging”, “neuroinflammation”, “microglia”, and “blood–brain barrier”. Candidate miRNAs were included when they met at least one of the following criteria: (i) direct evidence of an exercise-responsive, EV-associated change, or (ii) strong mechanistic evidence linking the miRNA to a pathway relevant to cognitive aging, even where EV-specific or exercise-specific data remain lacking. Building on this emerging literature, several recent works have summarized different facets of the field: broad narrative overviews have described exercise-responsive miRNAs and exosomes across skeletal muscle, cardiac, and metabolic tissues [15] and across metabolic, immune, cardiovascular, and nervous systems more generally [16]; mechanistic overviews have focused on EV biogenesis and its regulation by exercise, including a therapeutic perspective on neurodegenerative disease [17]; a recent commentary has explicitly raised the open question of whether exercise-induced, muscle-derived EVs actually deliver cognitive benefit to the brain, underscoring the same mechanistic gap this review addresses [18]; and disease-specific models have linked exercise-conditioned EVs to Alzheimer’s pathology [19] or examined the epigenetic regulatory mechanisms of exercise-derived EVs across organ systems [20]. None of these works, however, is centered specifically on cognitive aging in chronologically and biologically defined older adults, nor do they systematically distinguish exercise modalities or separate direct, EV-specific, exercise-responsive evidence from indirect, triangulated evidence for individual candidate miRNAs. The present review addresses this gap by narrowing the focus specifically to cognitive aging in older adults, rather than to a single disease entity or to physiological systems in general. We further distinguish between exercise modalities (aerobic, resistance, multicomponent, and mind–body interventions such as Tai Chi) rather than treating “exercise” as a single, undifferentiated exposure, and for each candidate EV-miRNA, we explicitly separate direct, EV-specific, exercise-responsive evidence from evidence that is only indirect or triangulated—a distinction we consider essential given the goal of eventually using these molecules as biomarkers of healthy brain aging.

## 2. Physical Activity and Brain Health in Older Adults

Physical activity is widely recognized as one of the most accessible non-pharmacological strategies supporting healthy brain aging. Regular exercise influences numerous biological pathways that are directly or indirectly related to cognitive functioning. In older adults, physical activity improves cardiovascular fitness, regulates blood pressure, supports glucose and lipid metabolism, increases muscle strength, improves balance, mood, and sleep quality, and modulates the inflammatory status of the organism. The term “older adults” refers to chronologically defined individuals aged approximately ≥60–65 years; this chronological definition is used together with, but is distinct from, the related concepts of biological aging (age-related decline in physiological function, which may diverge from chronological age), active aging, and healthy aging, as promoted by the World Health Organization [9,10]. Collectively, these factors can affect both the structure and function of the brain [10,11,21]. For this reason, physical exercise is increasingly considered both as a preventive strategy in older adults without cognitive impairment and a supportive rehabilitative approach for individuals with mild cognitive impairment or an elevated risk of dementia [22]. According to World Health Organization recommendations, older adults should engage in regular physical activity which includes aerobic exercise, muscle-strengthening activities, and components aimed at improving balance and motor coordination [10]. This approach is particularly important in aging, as it reduces the adverse consequences of sedentary behavior, supports functional independence, and is thought to indirectly lower the risk of cognitive decline. Physical activity therefore affects the brain not only through direct biological mechanisms, but also through improvements in general health status, motor function, metabolism, and immune regulation.

Table 1 presents four major forms of physical activity analyzed in the context of brain health in older adults, their potential cognitive effects, and the biological mechanisms that can mediate the protective influence of exercise on cognitive aging.

The cognitive effects of physical activity are likely the result of interactions between central and peripheral mechanisms. At the level of the central nervous system, regular physical activity supports hippocampal integrity and enhances functional connectivity. It also activates pathways associated with neurogenesis and synaptic plasticity, and strengthens neurotrophic signaling, including pathways related to brain-derived neurotrophic factor (BDNF) [24,25]. At the systemic level, physical activity promotes vascular and metabolic homeostasis, reduces chronic low-grade inflammation, and regulates immune responses, thereby influencing biological mechanisms involved in cognitive decline and the development of neurodegenerative processes [12,23]. The multidirectional effects of physical activity therefore indicate its important role in counteracting adverse changes associated with brain aging. However, despite extensive evidence supporting the importance of physical activity for maintaining cognitive and brain health, current research has several important limitations. Existing studies differ in terms of exercise modality, intensity, frequency, intervention duration, level of supervision, adherence to training protocols, cognitive assessment tools, and participant characteristics [13,23]. Moreover, relatively few studies combine the evaluation of behavioral outcomes with analyses of molecular biomarkers, neuroimaging findings, inflammatory markers, and indicators of biological brain aging. This gap is particularly relevant in the context of extracellular vesicle-associated microRNAs, which may represent a mechanistic link between peripheral adaptations to exercise and central processes such as neuroinflammation, synaptic plasticity, and neuronal resilience.

Future studies should therefore integrate structured exercise interventions with longitudinal assessments of EV-miRNA profiles, inflammatory and neurotrophic biomarkers, neuroimaging markers, and standardized cognitive testing in older adults. In this context, it is particularly important to clarify how biological signals generated during exercise are transmitted from peripheral tissues to the brain. One potential mechanism of this communication involves extracellular vesicles, which can transport bioactive molecules, including miRNAs, that participate in the regulation of neuroinflammation, synaptic plasticity, and neuronal responses to oxidative stress.

## 3. Extracellular Vesicles as Mediators of Exercise-Induced Inter-Organ Communication

Physical exercise induces a coordinated systemic response that extends far beyond the contracting skeletal muscle [36]. During and after an exercise session, multiple tissues release bioactive molecules into the circulation, including metabolites, cytokines, proteins, lipids, and nucleic acids [37]. Among these, exercise-induced “exerkines” have recently been shown to modulate autophagy across multiple organs, a process implicated in several hallmarks of aging, suggesting that exercise-responsive signaling molecules may influence systemic aging trajectories through mechanisms beyond classical endocrine or metabolic pathways [37]. These exercise-responsive factors may contribute to both acute homeostatic regulation and long-term adaptations to repeated training [36,37]. In this context, extracellular vesicles (EVs) have attracted increasing attention as potential carriers of exercise-induced molecular signals, as they can protect bioactive cargo from degradation and deliver it to local or distant recipient cells [38,39]. This concept is particularly relevant for aging research, where the beneficial effects of physical activity on brain health may depend not only on vascular or metabolic adaptations, but also on inter-organ molecular communication linking skeletal muscle, immune cells, vascular endothelium, and the central nervous system [38].

### 3.1. Definition and Classification of Extracellular Vesicles

Extracellular vesicles are lipid bilayer-enclosed particles released by cells into the extracellular environment. They are unable to replicate and can carry proteins, lipids, metabolites, mRNAs, microRNAs, and other non-coding RNAs. EVs are commonly detected in biological fluids such as blood, urine, saliva, and cerebrospinal fluid, making them relevant to the study of systemic communication in physiological and pathological conditions [40,41]. Importantly, EVs comprise a heterogeneous population rather than a single biological entity. They differ according to size, biogenesis, molecular composition, cellular origin, and functional properties [40,42].

Several classification approaches coexist, and each captures a different aspect of EV heterogeneity. Historically, EVs have often been classified into exosomes, microvesicles, and apoptotic bodies according to their presumed biogenesis and physical characteristics. Exosomes are generally associated with the endosomal pathway, whereas microvesicles arise through outward budding of the plasma membrane [40,43]. However, these categories cannot always be assigned reliably to vesicles isolated from complex biological fluids because the molecular and physical features used for classification may overlap, and the biogenesis of individual particles is often difficult to establish experimentally [41]. Consequently, current recommendations favor operational terminology based on experimentally supported characteristics, such as “extracellular vesicles” or “small extracellular vesicles”, rather than assigning a specific biogenetic category such as “exosomes” when their origin has not been demonstrated. The MISEV2023 guidelines emphasize the need for careful EV nomenclature, pre-analytical control, particle separation, characterization, and transparent reporting, especially when working with complex biofluids such as blood [41]. This absence of a single, settled classification scheme has been further compounded by the recent discovery of additional non-vesicular extracellular particles, such as exomeres and supermeres, which overlap in size and molecular cargo with classical EVs yet are structurally distinct—underscoring that EV nomenclature and classification remain areas of active methodological debate rather than settled consensus [44]. Classification can also be based on other experimentally accessible features. Physical schemes may distinguish EVs by size or density; biochemical schemes may describe populations enriched for particular membranes or cargo markers; and source-based schemes may define EVs by their cell or tissue of origin or by the condition under which they are generated. Proteomic fractionation has further demonstrated that vesicles of overlapping size can represent molecularly distinct subpopulations, supporting the use of marker combinations rather than size alone to characterize EV preparations [45]. In addition, context- or function-associated terms have been introduced for particular EV populations. Examples include large oncosomes, unusually large tumor-derived vesicles associated with oncogenic processes [46], and migrasomes, vesicular structures generated during cell migration and linked to the release of cytoplasmic material [47]. Such labels may be biologically informative, but they do not constitute a single universal functional taxonomy and should not be treated as interchangeable with size- or biogenesis-based categories. Each classification system has important limitations. Size ranges overlap substantially among EV populations, and estimates vary with techniques such as nanoparticle tracking analysis, electron microscopy, flow cytometry, and other particle-sizing methods. Similarly, density and molecular-marker profiles are not fully subtype-specific, while commonly used markers may be shared by EVs of different origins [46].

This methodological caution is particularly important in exercise studies. Physical activity acutely alters blood flow, shear stress, platelet activation, immune cell trafficking, metabolic status, hemoconcentration, and plasma volume, all of which may influence the concentration and composition of circulating particles [39]. Moreover, plasma-derived EV preparations can be contaminated by lipoproteins, protein aggregates, or other non-vesicular particles [41,48]. Therefore, studies investigating exercise-induced EVs should ideally combine several complementary methods, including particle analysis, protein-marker characterization, assessment of potential contaminants, and molecular cargo profiling [41]. Without such methodological rigor, changes in EV-associated miRNAs or proteins can be difficult to distinguish from changes in non-vesicular circulating molecules [39,41].

### 3.2. Exercise as a Stimulus for EV Release and Cargo Remodeling

A growing body of evidence indicates that acute exercise can alter the circulating EV profile [49,50]. Early work demonstrated that physical exercise induces a rapid release of small EVs into the circulation, with changes appearing during the aerobic phase of exercise and returning toward baseline during recovery [49]. Subsequent studies using proteomic and particle-based approaches supported the concept that exercise can transiently remodel the circulating EV pool and its molecular cargo [50]. Whitham et al. [50] showed that acute exercise modifies the EV-associated proteome in humans, and proposed EVs as a mechanism of tissue crosstalk during exercise.

The response of circulating EVs to exercise should not be interpreted solely as an increase in vesicle abundance. Exercise may also influence EV cellular origin, surface phenotype, molecular cargo, and biological activity. Exercise appears to alter EV abundance, surface-marker composition, cellular origin, and cargo density [50,51]. For example, blood-flow-restricted resistance exercise was shown to modify the surface profile and miRNA cargo of circulating EVs in healthy participants. In that study, several EV surface markers and miRNAs changed after exercise, and EVs isolated after the exercise session influenced skeletal-muscle precursor-cell proliferation in vitro [51]. These findings suggest that exercise-associated EVs can have biological activity, although their systemic relevance in vivo remains to be fully established.

Importantly, exercise-induced EV responses appear to depend on exercise modality, intensity, duration, timing of blood collection, and training status. Aerobic exercise, resistance exercise, high-intensity exercise, and blood-flow-restricted exercise may not generate the same EV signature. In addition, an EV profile observed immediately after exercise may differ from that detected several hours later. This temporal dimension is relevant for interpreting exercise-induced EV-miRNA changes, because miRNA cargo appears to reflect both acute stress responses and early recovery-related signaling [38,39].

### 3.3. Potential Cellular Sources of Exercise-Responsive EVs

Although skeletal muscle is a major candidate source of exercise-induced molecular signals, circulating EVs after exercise should not be attributed exclusively to muscle [39]. Exercise activates multiple tissues and cell types, including skeletal muscle fibers, vascular endothelial cells, platelets, leukocytes, erythrocytes, adipose tissue, and, possibly, cells associated with the nervous system [52]. Brahmer et al. [52] reported that platelets, endothelial cells, and leukocytes contribute to the exercise-triggered release of EVs into circulation, supporting the view that the circulating EV pool after exercise is heterogeneous and reflects a whole-body response rather than a muscle-only process.

Because circulating EV preparations obtained after exercise generally contain vesicles from multiple cellular sources, their potential role in exercise-related brain effects should be interpreted without presuming a skeletal-muscle origin. In human-plasma studies, attribution to muscle-derived EVs requires evidence based on tissue-associated markers, cell-specific enrichment, or experimental tracing; otherwise, the more precise term is “exercise-responsive circulating EVs” rather than “muscle-derived EVs” [39]. Muscle-derived EVs are biologically plausible mediators of muscle–brain communication, but plasma EVs measured in human studies usually represent a mixture of vesicles from different cellular sources [39,41]. Therefore, unless studies use tissue-specific markers, cell-specific EV isolation strategies, or experimental tracing approaches, the term “exercise-responsive circulating EVs” may be more accurate than “muscle-derived EVs” [41].

Nevertheless, muscle-derived EVs remain highly relevant to the concept of exercise-induced inter-organ communication. Contracting skeletal muscle is an endocrine and paracrine organ that releases myokines, metabolites, and vesicle-associated cargo [53]. Experimental evidence suggests that skeletal muscle-derived EVs are plausible contributors to exercise-induced inter-organ communication, and may participate in signaling toward distant organs, including the brain [54,55]. For instance, recent preclinical work demonstrated that muscle-derived small extracellular vesicles mediate exercise-induced cognitive protection in a mouse model of chronic cerebral hypoperfusion [56]. These vesicles enhanced synaptic plasticity through the delivery of miR-17/20a-5p and activation of hippocampal mTOR signaling [56]. Although such findings cannot yet be directly generalized to healthy older adults, they support the biological plausibility of EV-mediated muscle–brain signaling.

### 3.4. EVs as a Link Between Peripheral Exercise Adaptation and the Brain

The brain is not isolated from systemic physiological changes induced by exercise. Peripheral signals can influence brain function through humoral routes, immune modulation, endothelial signaling, metabolic adaptations, and neurovascular mechanisms [40,57]. EVs may contribute to this communication because they can interact with vascular barriers, including the blood–brain barrier, and can participate in bidirectional signaling between the central nervous system and peripheral organs [58]. 

In the context of cognitive aging, EVs provide a mechanistic framework linking exercise-induced peripheral adaptations with central processes such as neuroinflammation, microglial activation, oxidative stress, vascular integrity, and synaptic plasticity [50]. This does not imply that EVs are the sole mediators of exercise-related neuroprotection. Rather, they may act as one component of a broader adaptive network that also includes myokines, cytokines, metabolites, hormones, and changes in cerebral blood flow [50,57]. Their importance lies in their ability to transport complex molecular cargo, including miRNAs, which can regulate gene expression in recipient cells and potentially shape inflammatory or neuroplastic responses [40,42].

Evidence from older adults is still limited but directly relevant. In a study of older men, endurance-trained and sedentary participants showed distinct circulating exosomal miRNA profiles at baseline and after acute aerobic exercise [59]. The authors reported that regular exercise and acute exercise were associated with different exosomal miRNA signatures, and analyses suggested links with IGF-1 signaling [59], a pathway with well-established anabolic and adaptive roles in muscle, but also a central mediator of exercise-induced hippocampal neurogenesis, synaptic plasticity, and long-term potentiation, and one whose circulating levels decline with age in parallel with cognitive decline [60,61]. Although this study focused primarily on systemic and muscle-related pathways rather than cognitive outcomes, it demonstrates that training status may influence EV-miRNA responses in older individuals; however, as this study included only five endurance-trained and five sedentary participants, these findings should be regarded as preliminary and require confirmation in larger cohorts [59].

Taken together, current evidence supports the concept that EVs are responsive to exercise and may contribute to inter-organ communication [39,50]. However, in the specific context of cognitive aging, the field remains at an early stage [59]. Most available studies either examine EV responses to exercise without assessing brain outcomes, or investigate neuroprotective effects of exercise without detailed EV-miRNA profiling [39]. Therefore, exercise-responsive EVs should be considered a promising but still emerging molecular link between physical activity and brain health in older adults [39,50]. Establishing this link will require studies that combine standardized EV isolation and characterization, miRNA cargo analysis, markers of neuroinflammation, neurovascular function, and cognitive assessment [41].

## 4. miRNAs Carried by Extracellular Vesicles: Molecular Regulators of Inflammation and Neuronal Function

**Figure 1:**
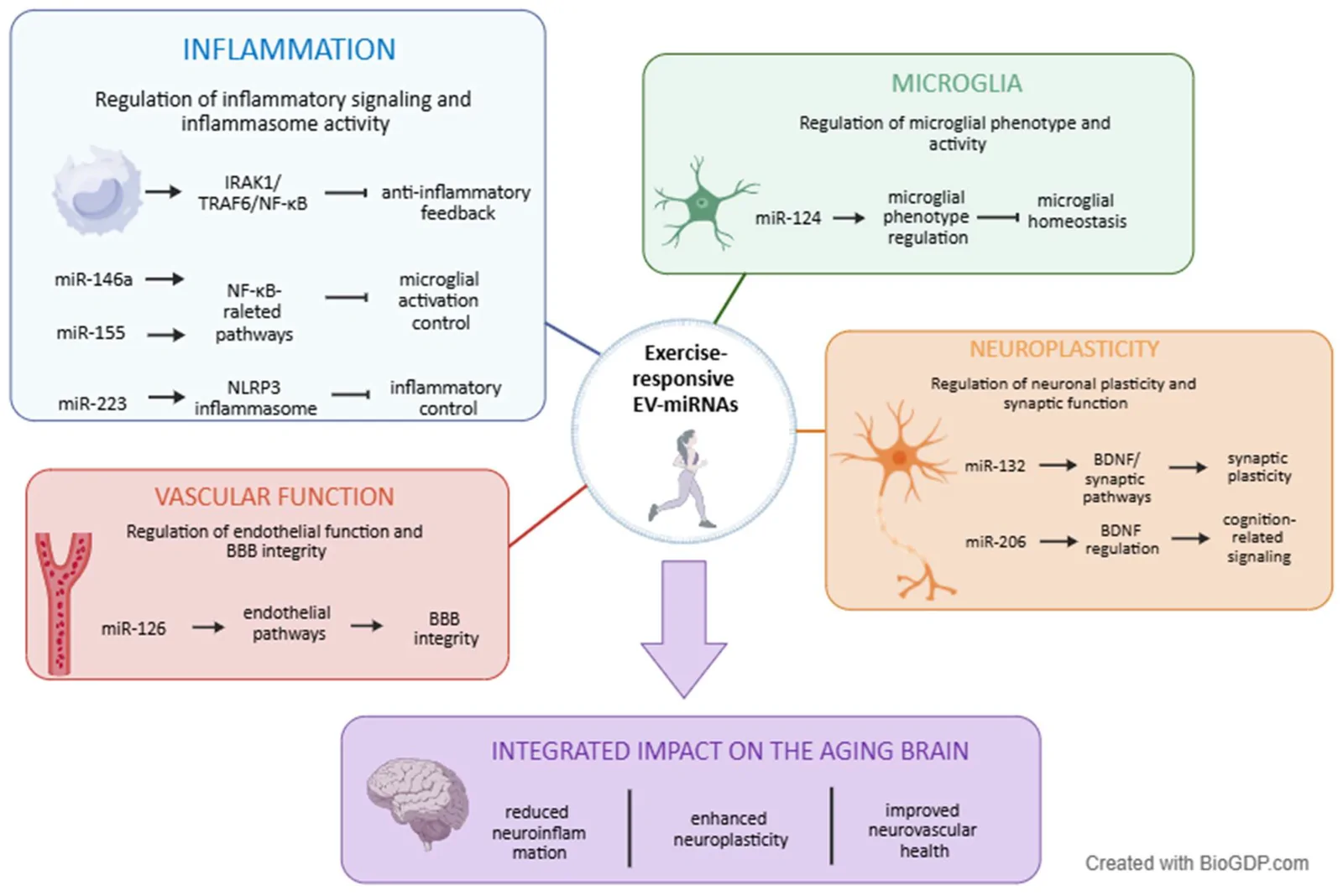
Exercise-responsive EV-miRNAs as regulators of molecular pathways involved in cognitive aging. Created with BioGDP.com [65].

MicroRNAs (miRNAs) are short non-coding RNAs that regulate gene expression mainly at the post-transcriptional level by interacting with target mRNAs and promoting mRNA degradation, deadenylation, or translational repression [62]. A single miRNA can regulate multiple transcripts, whereas a single transcript can be targeted by several miRNAs. This regulatory architecture allows miRNAs to fine-tune complex biological pathways rather than act as simple on–off molecular switches [62,63]. Mature miRNAs are processed from longer hairpin precursors, and for many loci, both strands of the hairpin give rise to functional mature products, conventionally distinguished by a “-3p” or “-5p” suffix (e.g., miR-146a-5p and miR-146a-3p); throughout this review, we follow the standard human (hsa-miR-) nomenclature and retain this designation where it was reported, noting explicitly the cases in which a cited study used a rodent or other non-human model, an in vitro/cell-culture model, or did not distinguish between precursor, mature, 3p, or 5p forms. This regulatory mode is particularly relevant in aging and exercise biology, where physiological adaptation depends on coordinated changes in inflammatory signaling, oxidative stress responses, vascular function, and neuronal plasticity [63,64]. From the perspective of cognitive aging, several EV-associated miRNAs emerge as biologically plausible candidates linking exercise-responsive extracellular vesicles with inflammatory regulation, neurovascular function, and neuronal plasticity. These candidates include miRNAs involved in NF-κB signaling, inflammasome regulation, microglial homeostasis, BDNF-related pathways, and endothelial function (Figure 1, Table 2).

When miRNAs are associated with extracellular vesicles, their biological significance may extend beyond intracellular gene regulation [85]. EVs can protect miRNAs from enzymatic degradation in biofluids and may facilitate their delivery to recipient cells [86]. In this way, EV-associated miRNAs may contribute to local and systemic communication between tissues [40]. In the context of exercise, EV-miRNAs are of particular interest because physical activity can alter both the number of circulating EVs and their molecular cargo [39,50]. However, it is important to distinguish between association and causality: the detection of an altered EV-miRNA profile after exercise does not necessarily prove that these miRNAs are functionally transferred to brain cells or directly responsible for cognitive benefits [41].

### 4.1. Exercise-Responsive EV-miRNAs as Coordinators of Systemic Adaptation

Exercise-induced EV-miRNAs may reflect the integrated response of multiple tissues, including skeletal muscle, endothelial cells, immune cells, platelets, and adipose tissue [50,52]. These miRNAs appear to participate in the regulation of pathways involved in tissue remodeling, inflammation, angiogenesis, metabolism, and cellular stress resistance [86]. In older adults, this is especially relevant because aging is associated with chronic low-grade inflammation, impaired vascular function, reduced mitochondrial resilience, and altered immune regulation, all of which may influence brain health and cognitive function [87,88].

Evidence from human studies supports the concept that aging and training status influence circulating EV-miRNA profiles [59]. In older men, sedentary and endurance-trained individuals showed distinct circulating exosomal miRNA signatures at baseline and after acute aerobic exercise. Pathway analysis suggested that exercise-regulated EV-associated miRNAs may modulate IGF-1 signaling and other adaptive pathways [59]. Although this study did not directly assess neuroinflammation or cognition, it demonstrates that EV-miRNA cargo is responsive to exercise and training status in older individuals, providing a mechanistic basis for further studies focused on brain aging [59].

From the perspective of cognitive aging, the most relevant EV-miRNAs are not necessarily those with the largest post-exercise fold change, but those that regulate biological processes known to contribute to brain aging. These include NF-κB-dependent inflammatory signaling, NLRP3 inflammasome activation, cytokine production, microglial activation states, blood–brain barrier integrity, oxidative stress responses, and neurotrophic signaling [89,90]. Therefore, the following sections focus on selected miRNAs that may represent candidate molecular links between exercise-responsive EVs and brain health.

### 4.2. EV-miRNAs Involved in Inflammatory Signaling

One of the most relevant inflammatory miRNAs in the context of exercise, aging, and neuroinflammation is miR-146a, although the evidence supporting this relevance is mechanistic and comes largely from immune and CNS injury models rather than from exercise studies [66]. It is commonly described as a negative feedback regulator of innate immune signaling, targeting components of Toll-like receptor and interleukin-1 receptor pathways, including IRAK1 and TRAF6, and thereby limiting downstream NF-κB activation and pro-inflammatory cytokine production [66,67]. EVs enriched in miR-146a have been proposed as a potential regulator of inflammatory signaling in these non-exercise contexts; however, direct evidence linking exercise-responsive EV-miR-146a changes with neuroinflammatory outcomes in older adults is still lacking [68]. Within this same non-exercise literature, miR-146a is further implicated in inflammasome-related signaling and microglial inflammatory responses [91]: experimental studies in microglial injury models show that miR-146a-5p-enriched microglial EVs can attenuate inflammatory injury by targeting pathways linked with HIF1α, mitochondrial reactive oxygen species, and NLRP3 inflammasome activation [69]. These findings support the mechanistic plausibility of EV-associated miR-146a as an anti-inflammatory regulatory signal, although they do not yet extend to exercise in older adults.

By contrast, miR-155 is generally associated with pro-inflammatory activation on the basis of evidence from immune-cell and microglial studies in neurodegenerative disease models rather than from exercise research [70]. In these models, miR-155 promotes inflammatory responses in immune cells and microglia, and increased miR-155 signaling has been linked with pathological inflammatory states in the central nervous system [71]. Reviews of this largely intracellular literature also describe miR-155 as an EV-associated molecule with potential relevance as a biomarker or therapeutic target in neurodegeneration [72], though this EV-related potential has not been examined in the context of exercise or aging.

The balance between miR-146a and miR-155 may therefore be biologically meaningful [70]. However, a shift toward reduced microglial activation is not intrinsically beneficial in every context, so this balance should be interpreted alongside the specific microglial program it engages rather than treated as a simple anti-inflammatory-is-better rule. Rather than focusing on a single miRNA, future studies could examine whether exercise shifts the EV-miRNA profile toward a less pro-inflammatory pattern; for example, by increasing anti-inflammatory miRNAs such as miR-146a or reducing excessive miR-155-associated signaling [41]. This ratio-based or network-based approach may be more informative than interpreting each EV-miRNA in isolation.

miR-21 is another miRNA with highly context-dependent effects, drawn from tissue-injury and fibrosis literature rather than from exercise or EV-specific studies. In peripheral tissues, miR-21 participates in tissue repair, fibrosis, immune regulation, and stress responses [75], and in the nervous system it has similarly been implicated in inflammatory responses and neuronal stress after injury. Whether EV-associated miR-21 contributes to neural inflammatory communication under physiological, exercise-responsive conditions remains untested; the available data only show that inflammatory conditions can alter miR-21 expression in neural cells and extracellular RNA profiles in disease or injury models [75].

miR-223 is particularly relevant to inflammasome regulation, based on evidence from immune and peripheral inflammatory models rather than from exercise studies in older adults [39,59]. In these models, it has been described as a regulator of NLRP3 inflammasome activity and macrophage inflammatory responses [74]. Because NLRP3 activation also contributes to age-related inflammation and neuroinflammatory signaling, EV-associated miR-223 represents a plausible, though still untested, candidate through which exercise-responsive EVs could influence inflammatory tone in older adults [39,87,89].

### 4.3. EV-miRNAs and Microglial Regulation

Microglia are central regulators of neuroimmune homeostasis [92]. During aging, microglia may become more reactive to peripheral and central inflammatory stimuli, contributing to a state often described as microglial priming [93]. This makes microglial signaling a critical target when considering how exercise-responsive EV-miRNAs might influence cognitive aging. EVs released by microglia can exert either protective or harmful effects depending on the inflammatory context and cargo composition, which reinforces the need to interpret EV-miRNA functions in a state-dependent manner [94,95].

miR-124 is one of the best-known miRNAs associated with microglial homeostasis, an association established through intracellular microglial signaling studies in neuroinflammatory disease models rather than through EV or exercise research. In these models, it has been described as a regulator of microglial activation and a potential therapeutic target: reduced miR-124 expression is associated with enhanced inflammatory activation, whereas restoration of miR-124 signaling may support a more homeostatic or anti-inflammatory microglial phenotype [73]. Whether EV-associated miR-124 contributes to exercise-induced adaptations in older adults has not yet been characterized.

The combined regulation of miR-124, miR-146a, and miR-155 may be especially important. miR-124 is associated with microglial homeostasis, miR-146a with negative feedback control of inflammatory signaling, and miR-155 with pro-inflammatory activation [66,70,73]. Exercise-induced EVs may theoretically influence neuroinflammation by changing the relative abundance of these miRNAs [50]. This hypothesis remains to be tested in longitudinal human studies combining exercise interventions, EV-miRNA profiling, neuroinflammatory biomarkers, and cognitive outcomes.

### 4.4. EV-miRNAs, BDNF-Related Signaling, and Synaptic Plasticity

Beyond inflammation, EV-associated miRNAs may influence neuronal function by regulating pathways involved in synaptic plasticity, neurotrophic signaling, and neuronal network remodeling [40,96]. This concept is particularly relevant to cognitive aging, as age-related decline is associated not only with neuronal loss but also with impaired synaptic maintenance and reduced plasticity [97].

Among neuronal miRNAs, miR-132 is one of the best-characterized regulators of activity-dependent synaptic plasticity based on evidence that comes entirely from intracellular, non-vesicular studies of neuronal signaling, not from EV or exercise research [76]. In these models, miR-132 expression is induced by neuronal activity and contributes to dendritic growth, synaptic remodeling, and memory-related processes through regulation of cytoskeletal and synaptic targets [76,77]. Whether exercise-responsive, EV-associated miR-132 reproduces any of these intracellular effects in older adults has not been tested; EV-mediated transport remains, at this point, a plausible but undemonstrated route by which such peripheral signals could reach neurons [96].

This mechanism is particularly relevant for exercise biology because physical activity is widely associated with neurotrophic and neuroplastic adaptations [98,99]. Although BDNF is often discussed as a soluble factor, EV-miRNA pathways may provide an additional layer of regulation [96]. In this model, exercise-induced changes in neurotrophic signaling could alter EV-miRNA cargo, which in turn may influence synaptic genes and neuronal connectivity. However, the extent to which this mechanism occurs in older humans after exercise remains unknown.

miR-206 is another miRNA of interest, on the basis of a non-vesicular animal model of neurodegeneration in which experimental inhibition of miR-206 enhanced BDNF expression and improved cognitive performance [78]. This evidence concerns intracellular miR-206 activity in a disease model—it says nothing about EV-associated or exercise-responsive miR-206, and whether exercise changes EV-miR-206 in a way that similarly affects BDNF signaling in older adults remains untested. A further complication is that miR-206 is a well-established myomiR in skeletal muscle biology [79], so any future EV-based finding in this area would first need to disentangle a muscle-adaptation signal from a neurotrophic one.

Other exercise-responsive or muscle-enriched miRNAs, including miR-1, miR-133a/b, miR-486. and miR-23a, may also contribute to systemic adaptation. These miRNAs are frequently discussed in relation to skeletal-muscle remodeling, anabolic signaling, mitochondrial regulation. and metabolic adaptation [84,100]. Their direct role in brain aging is less established, but they may indirectly influence brain health through improved muscle function, metabolic homeostasis, vascular health, and systemic inflammatory tone.

### 4.5. EV-miRNAs, Oxidative Stress, Vascular Function, and Blood–Brain Barrier Integrity

Cognitive aging is closely linked with vascular dysfunction, oxidative stress, and impaired blood–brain barrier integrity [90,101]. EV-associated miRNAs may participate in these processes by regulating endothelial activation, inflammatory adhesion molecules, angiogenic signaling, and oxidative stress responses [96]. Among candidate miRNAs, miR-126 is especially relevant on the strength of endothelial and vascular biology research, rather than exercise-specific EV studies [80]. In this vascular literature, miR-126 is described as an important regulator of cell survival, proliferation, migration, and intercellular communication, particularly in vascular contexts [81], and experimental models suggest it can attenuate neurovascular inflammation by reducing endothelial activation, limiting inflammatory signaling, and preserving blood–brain barrier integrity [80,101]. Whether exercise-responsive, EV-associated miR-126 reproduces these effects in older adults—and thereby link vascular adaptation to brain protection—has not been directly tested.

In addition to miR-126, miR-21, miR-210, and miR-29 family members may also contribute to pathways relevant to cognitive aging, including oxidative stress responses, angiogenesis, mitochondrial adaptation, and inflammatory remodeling [75,82,102]. miR-210 is a well-characterized hypoxia-responsive miRNA regulating endothelial function, mitochondrial metabolism, and vascular remodeling in ischemic and hypoxic models; its relevance to exercise-induced adaptation has been inferred from this hypoxia biology rather than demonstrated directly [102]. miR-21 and members of the miR-29 family, in turn, show highly context-dependent actions that vary by tissue type, physiological state, and disease condition in the studies from which they are drawn [75,82], so their behavior in exercise-responsive EVs in older adults cannot yet be predicted from this literature. Consequently, biological interpretation of exercise-responsive EV-miRNAs should consider the tissue of origin, recipient cell type, age, training status, and pathological context. Changes in individual miRNA abundance should, therefore, not be interpreted in isolation. Direct EV-specific evidence exists for miR-34a, though from aging and senescence models rather than exercise interventions: in mice, muscle-derived, alpha-sarcoglycan-positive circulating EVs have shown an age-related increase in miR-34a that induces senescence of bone marrow stromal cells, supporting its candidacy as an age- and muscle-associated EV cargo [83].

### 4.6. Candidate EV-miRNA Networks in Cognitive Aging

The available evidence is consistent with a network-based model in which exercise-responsive EV-miRNAs may influence cognitive aging through several interconnected mechanisms. First, anti-inflammatory miRNAs such as miR-146a and miR-223 appear to suppress NF-κB- and NLRP3-related signaling [66,74]. Second, microglia-related miRNAs such as miR-124, miR-146a, and miR-155 can regulate neuroimmune activation states [70,73]. Third, plasticity-related miRNAs such as miR-132 and miR-206 may influence BDNF-related signaling and synaptic remodeling [76,78]. Fourth, vascular miRNAs such as miR-126 may contribute to endothelial function and blood–brain barrier integrity [80,101].

This framework does not imply that all these miRNAs are consistently altered by exercise in older adults. Rather, they represent biologically plausible molecular candidates that connect EV-mediated communication with mechanisms known to participate in brain aging. Current reviews on exercise-induced EV-miRNAs emphasize that exercise type, intensity, duration, frequency, age, sex, and health status may all influence EV cargo [39,50]. Therefore, the same miRNA may show different responses depending on the exercise protocol and participant characteristics.

A major challenge for the field is to determine whether exercise-induced EV-miRNAs are merely biomarkers of systemic adaptation or active mediators of brain health. To establish causality, future studies should combine EV isolation and characterization, miRNA sequencing, or targeted miRNA profiling. Functional assays in recipient cells should also be integrated with clinical outcomes, including cognitive performance, neuroinflammatory markers, and functional status. In older adults, such integrated approaches are still rare, which makes EV-miRNAs promising but incompletely defined mechanisms linking physical activity with cognitive aging [50,59].

## 5. Neuroinflammation, Microglia, and Inflammaging as Targets of Exercise-Derived EV-miRNAs

Neuroinflammation is one of the central biological processes linking aging with progressive deterioration of brain function [103]. Under physiological conditions, inflammatory signaling in the central nervous system participates in tissue repair, synaptic remodeling, and host defense [92]. However, during aging, this response may become chronic, dysregulated, and less efficiently resolved [104]. This process is closely connected with inflammaging, defined as chronic, sterile, low-grade inflammation that develops with age and contributes to the pathogenesis of age-related diseases [104]. Inflammaging is not limited to the periphery; systemic inflammatory mediators may influence the brain through endothelial activation, altered blood–brain barrier function, immune-to-brain signaling, and changes in glial reactivity [101,103].

Microglia are key cellular mediators of this process. As resident immune cells of the central nervous system, microglia continuously survey the neural microenvironment and respond to changes in neuronal activity, tissue damage, protein aggregates, and inflammatory stimuli [92]. With aging, microglia may shift toward a more reactive and primed phenotype, characterized by increased sensitivity to secondary stimuli and altered production of cytokines, chemokines, reactive oxygen species, and other inflammatory mediators [93]. This age-associated microglial dysregulation may impair synaptic homeostasis, reduce neuroplasticity, and contribute to cognitive decline [92,93]. Recent single-cell transcriptomic studies suggest that hippocampal microglia undergo progressive transitions through intermediate cellular states during aging before acquiring more pronounced inflammatory phenotypes, highlighting the dynamic nature of age-associated microglial remodeling [105].

This inflammatory background provides a biologically plausible target for exercise-induced extracellular vesicle miRNAs. Regular exercise has been associated with modulation of neuroinflammatory pathways, improved cognitive performance, altered microglial and astrocytic activation states, and preservation of blood–brain barrier integrity in both experimental models and human studies [99,106]. In the context of neurodegenerative disorders, consistent physical exercise has been associated with altered neuroinflammatory pathways, improved cognitive outcomes, modulation of microglial and astrocytic activation states, and preservation of blood–brain barrier integrity [103]. However, the molecular mediators responsible for translating peripheral exercise adaptations into central neuroimmune effects remain incompletely defined. EV-miRNAs may represent one such mechanism because they can carry regulatory signals from exercise-responsive tissues to recipient cells involved in immune, vascular, and neural regulation [50].

The potential relevance of EV-miRNAs to neuroinflammation is supported by the fact that microglia themselves both release and respond to extracellular vesicles. Microglia-derived EVs can exert heterogeneous effects depending on the activation state of the donor cells and the molecular composition of their cargo. In homeostatic or reparative contexts, EVs may support intercellular communication, debris clearance, and inflammation resolution [95]. In contrast, under pathological inflammatory conditions, EVs may propagate neuroinflammatory signals [96]. Experimental studies have demonstrated that extracellular vesicles released under neuroinflammatory conditions can transfer miRNAs and other regulatory molecules capable of modulating microglial activation and inflammatory signaling. These findings suggest that EV cargo may actively shape neuroimmune responses rather than merely reflect disease status [95,96].

From this perspective, exercise-derived EV-miRNAs may influence cognitive aging by shifting neuroimmune signaling away from a chronic pro-inflammatory state toward a more regulated, adaptive response. Candidate miRNAs discussed in the previous section, including miR-146a, miR-124, miR-155, miR-21, and miR-223, are particularly relevant because they are connected with innate immune signaling, NF-κB regulation, inflammasome activity, and microglial activation [70]. An exercise-induced increase in EV-associated miRNAs with anti-inflammatory or inflammation-resolving properties, such as miR-146a or miR-124, could theoretically attenuate excessive microglial reactivity. Conversely, reduced delivery of pro-inflammatory miRNAs such as miR-155 could contribute to a less inflammatory neuroimmune profile. This interpretation remains hypothetical in older humans, but it provides a testable mechanistic framework for future exercise-intervention studies.

A second mechanism involves the interaction between neuroinflammation and oxidative stress [103]. Aging is accompanied by progressive mitochondrial dysfunction and impaired mitochondrial quality control. Increased production of reactive oxygen species (ROS) may further promote inflammatory signaling and amplify microglial activation [88]. In neurodegenerative conditions, oxidative stress and neuroinflammation are closely interrelated, and may contribute to synaptic dysfunction, neuronal injury, and impaired cognitive performance [103]. EV-miRNAs may participate in this network by regulating genes involved in redox signaling, mitochondrial stress responses, inflammatory cytokine production, and endothelial activation [96]. Therefore, exercise-induced remodeling of EV-miRNA cargo may contribute not only to immunomodulation, but also to improved cellular stress resilience within the neurovascular and neuroimmune environment.

The blood–brain barrier is another important interface through which systemic exercise-induced signals may influence brain aging [101]. Age-related inflammation and vascular dysfunction can compromise barrier integrity, allowing peripheral inflammatory mediators to affect the central nervous system more strongly [101,103]. Exercise may support neurovascular health by improving endothelial function, reducing inflammatory activation, and promoting vascular homeostasis [99]. EV-miRNAs with vascular and endothelial relevance, such as miR-126, may therefore be particularly important in linking peripheral exercise adaptations with brain protection [80]. EV-miRNAs may modulate endothelial adhesion molecules, inflammatory signaling, and barrier permeability. Through these effects, they could indirectly influence microglial activation and neuroinflammatory tone [80,96].

Recent preclinical findings provide stronger support for the connections between exercise, EVs, and microglial function [107]. In an Alzheimer’s disease mouse model, exercise was reported to alleviate cognitive dysfunction through skeletal muscle-derived extracellular vesicles that enhanced amyloid plaque clearance by microglia. Mechanistically, miR-378a-3p was identified as a major EV cargo promoting disease-associated microglial activation through regulation of PI3K p110α-dependent lipid metabolism [107]. This finding complicates a purely anti-inflammatory reading of exercise-responsive EV-miRNAs: here, a favorable outcome (amyloid plaque clearance) was associated with increased, disease-associated microglial activation rather than its suppression, in contrast to the anti-inflammatory framing emphasized for miR-146a, miR-155, and miR-124 elsewhere in this review. Therefore, whether a given shift in microglial activation state is beneficial or harmful cannot be inferred from its direction alone; it depends on the specific microglial program engaged, the brain region, and the disease or aging stage in question. Although these results cannot be directly extrapolated to healthy older adults, they strengthen the biological plausibility of EV-mediated muscle–brain communication in cognitive aging while cautioning against a one-directional model of microglial benefit.

At the same time, it is important to avoid oversimplification. Exercise-induced benefits for the aging brain are unlikely to be mediated by a single miRNA, a single EV subtype, or a single source tissue. Instead, EV-miRNAs should be considered as part of a broader adaptive network that includes myokines, cytokines, metabolites, hormones, neurotrophic factors, changes in cerebral blood flow, and improvements in systemic metabolic health [50,57]. Moreover, EV effects may be context-dependent. The same miRNA may have protective, compensatory, or maladaptive effects depending on the donor cell, recipient cell, inflammatory state, disease stage, and age-related biological environment [75]. This is especially relevant in older adults, in whom immunosenescence, comorbidities, medication use, and baseline physical activity may modify EV-miRNA responses to exercise.

Overall, this section has highlighted three main, interrelated mechanisms through which exercise-responsive EV-miRNAs may influence brain aging: regulation of microglial activation states, modulation of oxidative stress, and preservation of blood–brain barrier integrity. Neuroinflammation, microglial activation, and inflammaging represent biologically meaningful targets of exercise-derived EV-miRNAs. The current evidence supports the concept that physical activity remodels circulating EV cargo, including miRNAs capable of regulating inflammatory, oxidative, vascular, and neuroplastic pathways, although direct causal evidence in humans remains limited [50,107]. These EV-miRNAs may contribute to the attenuation of chronic neuroimmune activation and support mechanisms important for cognitive resilience. Nevertheless, direct evidence in older adults remains limited [59]. Future studies should determine whether changes in exercise-responsive EV-miRNAs are associated with measurable reductions in neuroinflammatory biomarkers, improved microglial-related signaling, preserved blood–brain barrier function, and better cognitive performance. Such studies would help clarify whether EV-miRNAs are only biomarkers of exercise adaptation or active molecular mediators of brain health in aging.

## 6. Biomarker and Therapeutic Potential of EV-miRNAs

Exercise-responsive EV-associated miRNAs satisfy the basic requirement of analytical validity for a candidate biomarker: they are detectable in peripheral biofluids, are relatively protected from degradation when enclosed, or are associated with vesicular structures [85]. Whether their abundance reliably tracks exercise exposure, aging, or cognitive status (biological association) is far less established, and needs to be confirmed candidate-by-candidate rather than assumed from detectability alone. Unlike single soluble proteins, EV-miRNAs may, in principle, carry information about coordinated regulatory networks spanning inflammation, vascular function, oxidative stress, and neurotrophic signaling [50,108]. However, this integrative potential is conceptual, and no EV-miRNA profile has yet been validated as a monitoring tool for biological responses to exercise in older adults.

From a preventive and rehabilitative perspective, EV-miRNAs may be useful as indicators of individual responsiveness to exercise. Older adults differ substantially in baseline inflammatory status, physical fitness, comorbidities, medication use, metabolic health, and cognitive reserve [88]. As a result, the same training program may not induce the same molecular or functional response in every individual [109]. If such a biological association is confirmed prospectively, EV-miRNA signatures could, in principle, help to identify whether a given exercise intervention induces a favorable biological shift; for example, toward reduced inflammatory signaling, improved endothelial function, enhanced neurotrophic support, or better stress resilience. At the level of predictive validity, they could eventually support a more personalized approach to exercise prescription in gerontology and neurorehabilitation [110].

Several lines of evidence support the feasibility of this concept, although direct data from cognitively healthy older adults remain limited. In older individuals, circulating exosome-like vesicle markers and miRNAs associated with skeletal-muscle health appear altered with age, and may partially increase following resistance exercise training [59]. This suggests that at least some age-related changes in EV-associated communication are modifiable by exercise. However, these findings primarily concern skeletal-muscle adaptation rather than brain outcomes, and therefore should be interpreted as evidence of trainability of EV-related signals rather than direct evidence of cognitive protection.

A more brain-oriented proof of concept comes from studies of neuron-derived extracellular vesicles [111]. Neuron-derived EVs isolated from blood have been proposed as accessible biomarkers of central nervous system processes, including neurodegenerative disease-related changes. In Alzheimer’s disease, blood-derived neuron-enriched EVs may reflect molecular alterations that are otherwise difficult to assess without cerebrospinal fluid sampling or neuroimaging [111]. A randomized exercise trial in patients with mild to moderate Alzheimer’s disease demonstrated the feasibility of using plasma neuron-derived EVs to monitor exercise-associated molecular changes. Delgado-Peraza et al. reported that a 16-week exercise intervention altered neuroprotective proteins, including proBDNF, BDNF, and humanin, within neuron-derived EVs isolated from plasma. Although this study analyzed protein cargo rather than miRNAs and included individuals with Alzheimer’s disease rather than cognitively healthy older adults, it provides an important proof of concept that circulating EVs may capture brain-related molecular responses to exercise [112].

EV-miRNAs may also have potential as disease-risk or disease-progression biomarkers, though the supporting evidence again comes from a different context than the one addressed here: cross-sectional case–control comparisons in diagnosed Alzheimer’s disease, rather than exercise studies in cognitively healthy older adults [113]. In this disease-comparison literature, EV-enriched miRNAs showed stronger biological associations with dementia status than total serum miRNAs in some studies, suggesting that vesicle-associated miRNA profiling may improve signal specificity compared with unfractionated circulating miRNAs [114]. Extending this reasoning to cognitive aging is plausible in principle, since subtle neuroinflammatory, vascular, and synaptic changes may begin years before clinically apparent cognitive impairment [115]. However, whether exercise modifies these same EV-miRNAs, and whether doing so carries any predictive value for future cognitive trajectories in healthy older adults, remains an untested hypothesis rather than a demonstrated finding.

However, the biomarker potential of EV-miRNAs should not be overstated. At present, EV-miRNAs should be considered promising research biomarkers rather than clinically validated tools [41,108]. Major obstacles include heterogeneity of EV populations and uncertainty regarding their cell or tissue of origin. Additional challenges include variability in sample processing, differences in EV isolation methods, lack of standardized normalization strategies for miRNA analysis, and limited reproducibility across studies [41]. These limitations are especially important in exercise research, where hydration status, plasma volume shifts, platelet activation, timing of blood collection, exercise intensity, and recent physical activity may all influence circulating EV measurements [43,50]. Current MISEV2023 recommendations emphasize transparent reporting, careful nomenclature, appropriate EV characterization, and control of pre-analytical and analytical variables, all of which are necessary before EV-miRNAs can be translated into reliable biomarkers [41].

In addition to their biomarker potential, EV-miRNAs may have therapeutic relevance. From a miRNA-directed perspective, therapeutic strategies may involve either supplementation of beneficial miRNAs using miRNA mimics or suppression of disease-associated miRNAs using antisense inhibitors, although delivery, tissue specificity, and off-target effects remain major barriers to translation. Conceptually, exercise-derived EVs could inspire future strategies aimed at mimicking selected molecular benefits of physical activity or delivering regulatory molecules [50,86]. Because EVs can transport miRNAs and other bioactive cargo, engineered or naturally derived EVs might be used in the future to modulate neuroinflammation, enhance neuroprotection, support blood–brain barrier integrity, or promote synaptic plasticity [86]. Preclinical studies already suggest that EV-based approaches can influence amyloid pathology, microglial activation, astrogliosis, and synaptic markers in Alzheimer’s disease models, supporting the plausibility of EV-mediated neuroprotection [107].

Nevertheless, therapeutic translation remains much more challenging than biomarker development. EV-based interventions require careful control of source cells, cargo composition, dosing, biodistribution, delivery route, immune compatibility, safety, and long-term effects [86]. The same EV-miRNA may have different biological consequences depending on the recipient cell type, disease context, and inflammatory state [86,108]. Moreover, exercise-induced EVs are not a single therapeutic entity, but a heterogeneous mixture of vesicles derived from multiple tissues [41,50]. Therefore, the therapeutic use of exercise-derived EVs or EV-miRNAs should currently be regarded as an experimental concept rather than a near-term clinical application.

Methodological barriers currently limit the use of EV-miRNA assays in routine clinical practice. EV isolation methods differ in yield, purity, and susceptibility to co-isolation of lipoproteins and protein aggregates, and no single marker can define a specific EV subtype. In addition, miRNA quantification is affected by extraction efficiency, normalization strategy, and platform-specific bias. For these reasons, EV-miRNA measurements should currently be regarded as research tools rather than clinical tests [39].

For the purposes of healthy aging, the most realistic short- and medium-term application of EV-miRNAs is likely biomarker-based monitoring rather than direct therapy. Future studies could evaluate whether changes in selected EV-miRNAs after acute or chronic exercise correlate with improvements in cognitive performance, executive function, memory, gait speed, balance, muscle strength, inflammatory markers, or neurovascular indicators. Such an approach would be particularly useful if EV-miRNA signatures could distinguish responders from non-responders. They could also help identify individuals who require modified exercise prescriptions [109]. Recent studies in older adults already show that acute exercise can modulate immune-cell subsets, cytokines, and EV profiles, supporting the need to integrate EV analysis with immunological and functional outcomes in aging research [59].

Overall, EV-miRNAs represent a promising interface between molecular exercise physiology and clinical geroscience. Their greatest value may lie in their ability to capture multi-system adaptation to physical activity, including inflammatory, vascular, muscular, and potentially brain-related responses [50]. The current literature is too limited and heterogeneous to support conclusions about therapeutic efficacy in cognitive aging, and the use of exercise-derived EVs or EV-miRNAs as treatments should therefore be considered as a hypothesis-generating concept. Future work will require standardized EV characterization, rigorous preclinical validation, dose–response and biodistribution studies, and appropriately designed human trials before any therapeutic claims can be substantiated [41]. In this sense, EV-miRNAs should be viewed not as isolated molecular markers, but as components of an integrated biological network linking exercise, neuroinflammation, and brain health in older adults.

## 7. Conclusions and Future Perspectives

Physical activity is one of the most important non-pharmacological strategies supporting healthy aging and preserving cognitive function in older adults. Although its beneficial effects have traditionally been explained by improved cardiovascular fitness, metabolic regulation, cerebral perfusion, and neurotrophic support, increasing attention is now being directed toward molecular mediators of exercise-induced inter-organ communication. Among these, extracellular vesicles and their miRNA cargo represent a promising mechanism through which peripheral adaptations to exercise may influence brain health [116].

Exercise-responsive EV-miRNAs may provide a molecular link between skeletal muscle, immune cells, vascular endothelium, and the central nervous system [50,117]. By regulating inflammatory signaling, oxidative stress responses, endothelial function, blood–brain barrier integrity, and neuroplasticity-related pathways, EV-miRNAs may contribute to the modulation of neuroinflammation and microglial activity during aging. This is particularly relevant in the context of inflammaging, where chronic low-grade inflammation and altered immune regulation are considered important contributors to cognitive decline [118].

Several miRNAs emerge as biologically plausible candidates linking exercise-induced EV remodeling with neuroimmune and neuroplastic processes. These miRNAs include miR-146a, miR-124, miR-155, miR-21, miR-223, miR-126, miR-132, and miR-206. They are involved in pathways associated with NF-κB signaling, inflammasome activation, microglial regulation, BDNF-related signaling, vascular homeostasis, and synaptic plasticity. However, their role should be interpreted within a network-based framework rather than as isolated molecular markers, because the biological effect of each miRNA depends on the donor cell, recipient cell, physiological context, age, inflammatory state, and exercise stimulus [119].

From a translational perspective, EV-miRNAs may be particularly valuable as biomarkers of biological responsiveness to exercise interventions [41]. In older adults, such biomarkers could help determine whether a given training program induces favorable molecular changes. These changes may involve inflammation, vascular function, neuroprotection, and cognitive resilience. This may support the development of more personalized exercise strategies in prevention, rehabilitation, and healthy aging [110]. At present, however, EV-miRNAs should be regarded primarily as promising research tools rather than clinically validated biomarkers [41].

Future studies should integrate exercise intervention protocols with standardized EV isolation and characterization, targeted or high-throughput miRNA profiling, neuroinflammatory biomarkers, and cognitive assessment [41]. Particularly valuable would be longitudinal studies in older adults that compare different exercise modalities, such as aerobic, resistance, and multicomponent training, and examine whether EV-miRNA signatures correlate with changes in memory, executive function, functional independence, and markers of brain health [110]. Such studies would help clarify whether exercise-induced EV-miRNAs are only indicators of systemic adaptation or active mediators of cognitive benefits.

In conclusion, exercise-derived EV-miRNAs represent an emerging and biologically plausible mechanism linking physical activity with neuroinflammation, microglial regulation, and cognitive aging. Although the field is still developing, this concept provides a valuable framework for understanding how lifestyle-based interventions may influence brain health at the molecular level. Further research in older populations may contribute to the identification of EV-miRNA signatures that reflect, predict, or even mediate the protective effects of physical activity on the aging brain.

## Figures and Tables

**Table 1:** Selected forms of physical activity and their relevance to brain health and cognitive function in older adults.

Type of Physical Activity	Intervention Characteristics	Cognitive Effects	Potential Biological Mechanisms	References
Aerobic training	Includes walking, brisk walking, cycling, swimming, and other forms of endurance exercise that engage large muscle groups.	Improvement in spatial memory, executive functions, and global cognitive performance; increased hippocampal volume; improved functional connectivity of neural networks.	Improvement in cardiorespiratory fitness, increased cerebral perfusion, enhanced vascular function, and activation of neurotrophic pathways, including BDNF-dependent mechanisms.	[12,23,24,25]
Resistance training	Includes strength exercises performed using body weight, resistance bands, free weights, or exercise machines.	Supports executive functions, selective attention, and conflict-resolution ability; contributes to the maintenance of functional independence in older adults.	Increased muscle strength and muscle mass, improved glucose metabolism, counteraction of sarcopenia, and possible modulation of neurotrophic, inflammatory, and metabolic factors.	[26,27,28]
Multicomponent training	Combines elements of aerobic, resistance, balance, coordination, and functional training.	Improvements in global cognitive performance, mobility, balance, and independence.	Integration of cardiovascular, metabolic, muscular, sensorimotor, and cognitive mechanisms; engagement of attention, motor planning, and executive control.	[13,22,29]
Tai Chi and Qigong	Mind–body forms of physical activity combining gentle movement, postural control, breathing regulation, attentional focus, coordination, and relaxation.	Improvement in executive functions, as well as balance, postural control, and physical performance.	Combination of physical activity with attentional regulation, stress reduction, breathing control, and motor coordination; modulation of inflammatory processes, oxidative stress, and the stress-response axis.	[30,31]
Yoga	Mind–body practice combining physical postures (asanas), controlled breathing (pranayama), and meditative/mindfulness components, typically delivered in structured group or individual sessions.	Improvements in attention, processing speed, memory, and executive function, in older adults both with and without mild cognitive impairment.	Modulation of the autonomic nervous system and stress-response axis, reduction in inflammatory and oxidative stress markers, and possible effects on cortical thickness and gray-matter volume.	[32,33,34,35]

**Table 2:** Candidate exercise-responsive EV-associated miRNAs potentially involved in cognitive aging and brain health. Labels in parentheses indicate the strength of the evidentiary chain linking exercise to EV cargo change to brain/neurovascular delivery to cognitive outcome: “triangulated” = no single study directly connects exercise, EV association, and the proposed pathway; “partial-direct” = one link in this chain (typically the exercise-responsive EV association itself) is directly demonstrated, while the remaining links (brain delivery, cognitive outcome) remain inferred.

miRNA (Evidence Chain)	Main Molecular Targets/Pathways	Biological Relevance to Cognitive Aging	Potential Role in Exercise-Induced Adaptation	Current Evidence and Limitations	Reference
miR-146a (triangulated)	IRAK1, TRAF6, NF-κB signaling, inflammatory feedback regulation	Regulation of innate immune responses, microglial activation, and neuroinflammatory tone	May contribute to attenuation of excessive inflammatory signaling after exercise by promoting a more regulated immune phenotype	Strong mechanistic evidence in inflammatory and CNS models; direct evidence in exercise-induced EV-miRNAs in older adults remains limited	[66,67,68,69]
miR-155 (triangulated)	NF-κB signaling, inflammatory cytokine pathways, immune cell activation	Associated with pro-inflammatory responses, microglial activation, and neuroinflammatory states	Exercise-related modulation could influence the balance between adaptive and excessive inflammatory signaling	Mainly supported by neuroinflammation and immune studies; exercise-specific EV evidence is insufficient	[70,71,72]
miR-124 (triangulated)	Microglial differentiation pathways, inflammatory regulators, neuronal homeostasis pathways	Maintains microglial homeostatic phenotype and limits excessive activation	May support anti-inflammatory microglial responses induced by physical activity	Evidence mainly from CNS and neuroinflammatory models; role in circulating exercise-responsive EVs requires investigation	[73]
miR-223 (triangulated)	NLRP3 inflammasome regulation, inflammatory signaling, macrophage responses	Regulation of inflammasome activity relevant to inflammaging and neuroinflammation	May contribute to reduced inflammatory activation through modulation of immune-derived EV cargo	Evidence for EV-mediated effects exists mainly in immune models rather than aging exercise studies	[74]
miR-21 (triangulated)	PTEN/PI3K-Akt signaling, inflammatory pathways, tissue-repair responses	Context-dependent effects in inflammation, cellular stress, and neuronal injury	May reflect adaptive stress responses induced by exercise, although effects depend on cellular origin and environment	Widely studied miRNA; biological effects are highly context-dependent	[75]
miR-132 (triangulated)	CREB signaling, BDNF-related pathways, synaptic remodeling	Key regulator of neuronal plasticity, dendritic growth, and memory-related processes	May represent a molecular link between exercise-induced neurotrophic signaling and synaptic adaptation	Strong neuronal evidence; direct regulation through exercise-derived EVs in older adults remains unclear	[76,77]
miR-206 (partial-direct)	BDNF regulation, muscle differentiation pathways, myogenic signaling	Potential involvement in cognitive decline through modulation of neurotrophic pathways	May reflect muscle–brain communication because it is both exercise-responsive and muscle-enriched	Interpretation of circulating EV-miR-206 is complicated by its muscle origin and pleiotropic effects	[78,79]
miR-126 (triangulated)	VEGF signaling, endothelial function, vascular homeostasis, adhesion molecules	Supports neurovascular integrity and blood–brain barrier function	May contribute to exercise-induced vascular adaptations supporting brain health	Strong vascular evidence; limited direct evidence connecting EV-miR-126 with cognitive aging	[80,81]
miR-29 family (triangulated)	Extracellular matrix remodeling, fibrosis-related pathways, cellular senescence pathways	Relevant to vascular aging, extracellular matrix changes, and age-related remodeling	May participate in systemic adaptation to exercise-induced tissue remodeling	Effects vary strongly depending on tissue and pathological context	[82]
miR-34a (partial-direct)	p53 signaling, apoptosis, cellular senescence pathways	Associated with aging, neuronal stress responses, and cognitive decline	Exercise-associated modulation may influence cellular stress resilience	Mostly studied in aging and neurodegeneration; limited EV-specific exercise evidence	[83]
miR-1 miR-133a/b miR-486 (partial-direct)	Muscle remodeling, mitochondrial adaptation, anabolic signaling pathways	Indirect relevance through preservation of muscle function, metabolism, and systemic health	Potential mediators of muscle–brain communication following exercise	Primarily myomiRs; direct contribution to cognitive aging remains hypothetical	[72,79,84]

Where established, the cellular/tissue origin of each candidate EV-miRNA is: miR-146a, miR-155, miR-124, and miR-223—immune and microglial cells; miR-132, miR-206, and the myomiRs—skeletal muscle and/or neurons; miR-126, miR-210, and the miR-29 family—vascular endothelium; miR-34a—skeletal muscle. For most candidates, the exact cellular source of the EV-associated (rather than intracellular) pool has not been directly established. Abbr.: EV, extracellular vesicles BBB, blood–brain barrier; NF-κB, nuclear factor kappa-light-chain-enhancer of activated B cells; NLRP3 NOD-, LRR- and pyrin domain-containing protein 3; BDNF, brain-derived neurotrophic factor.

## Data Availability

No new data were created or analyzed in this study. Data sharing is not applicable to this article.

## Abbreviations

Following abbreviations are used in this manuscript:

AD	Alzheimer’s disease
BBB	Blood–brain barrier
BDNF	Brain-derived neurotrophic factor
CNS	Central nervous system
CREB	cAMP response element-binding protein
EV	Extracellular vesicle
EV-miRNA	Extracellular vesicle-associated microRNA
HIF-1α	Hypoxia-inducible factor 1 alpha
IGF-1	Insulin-like growth factor 1
IL-1R	Interleukin-1 receptor
IRAK1	Interleukin-1 receptor-associated kinase 1
miRNA	MicroRNA
MISEV	Minimal Information for Studies of Extracellular Vesicles
NF-κB	Nuclear factor kappa B
NLRP3	NOD-, LRR- and pyrin domain-containing protein 3
PI3K	Phosphoinositide 3-kinase
PTEN	Phosphatase and tensin homolog
ROS	Reactive oxygen species
sEV	Small extracellular vesicle
TNF	Tumor necrosis factor
TRAF6	TNF receptor-associated factor 6
VEGF	Vascular endothelial growth factor
